# Goji Berry (*Lycium barbarum*) Supplementation during Pregnancy Influences Insulin Sensitivity in Rabbit Does but Not in Their Offspring

**DOI:** 10.3390/ani12010039

**Published:** 2021-12-25

**Authors:** Gabriele Brecchia, Majlind Sulce, Giulio Curone, Olimpia Barbato, Claudio Canali, Alessandro Troisi, Albana Munga, Angela Polisca, Stella Agradi, Maria Rachele Ceccarini, Daniele Vigo, Alda Quattrone, Susanna Draghi, Laura Menchetti

**Affiliations:** 1 Department of Veterinary Medicine, University of Milano, Via dell’Università 6, 26900 Lodi, Italy; gabriele.brecchia@unimi.it (G.B.); giulio.curone@unimi.it (G.C.); daniele.vigo@unimi.it (D.V.); susanna.draghi@unimi.it (S.D.); 2 Faculty of Veterinary Medicine, Agricultural University of Albania, Rr Paisi Vodica, Koder, 1029 Kamez, Albania; msulce@ubt.edu.al (M.S.); amunga@ubt.edu.al (A.M.); 3 Department of Veterinary Medicine, University of Perugia, Via San Costanzo 4, 06121 Perugia, Italy; olimpia.barbato@unipg.it (O.B.); claudio.canali@unipg.it (C.C.); angela.polisca@unipg.it (A.P.); alda.quattrone@hotmail.it (A.Q.); 4 Department of Veterinary Medicine, University of Camerino Via Circonvallazione 93/95, 62024 Matelica, Italy; alessandro.troisi@unicam.it; 5 Department of Pharmaceutical Sciences, University of Perugia, Via Fabretti, 48, 06123 Perugia, Italy; mariarachele.ceccarini@unipg.it; 6 Department of Agricultural and Food Sciences, University of Bologna, Viale G. Fanin 44, 40137 Bologna, Italy; laura.menchetti@unibo.it

**Keywords:** intravenous glucose tolerance tests, insulin resistance, hypoglycaemic effect, foetal metabolic programming, nutraceuticals, gestational diabetes

## Abstract

**Simple Summary:**

Diabetes mellitus is a disorder affecting millions of people worldwide. History of gestational diabetes is a proven risk factor, while diet may be a strategy for its prevention. Goji berry is the fruit of *Lycium barbarum* and a traditional medicinal herb with potential antidiabetic and hypoglycemic effects. This study evaluated the effect of two doses of Goji berry dietary supplementations on insulin sensitivity in rabbit does during pregnancy by using fasting and intravenous glucose tolerance test-derived indices. A long-term effect on the offspring was also hypothesised. The rabbit was a good experimental model for this study of insulin sensitivity as the tolerance test was feasible and sensitive to dietary modifications. The higher dose of Goji berry supplementation reduced the maximum glucose concentration after bolus administration, suggesting an improvement in the insulin response. Conversely, the present study could not support the effect of maternal diet on the adult offspring’s insulin sensitivity. The use of nutraceuticals as well as the hypothesis of foetal programming of metabolic diseases deserve further study.

**Abstract:**

This study investigated the effects of Goji berry (*Lycium barbarum)* dietary supplementation during pregnancy on insulin sensitivity of rabbit does and their offspring. Starting from two months before the artificial insemination, 75 New Zealand White does were fed only commercial standard diet (C) or supplemented with 1% (G1) and 3% (G3) of Goji berries. Their offspring received a standard diet but kept the nomenclature of the mother’s group. Fasting and intravenous glucose tolerance test-derived indices were estimated at 21 days of pregnancy on rabbit does and at 90 days of age on the offspring. No difference was found in the fasting indices, while the diet modulated the response to glucose load of rabbit does. In particular, G3 group had the lowest glucose concentrations 5 min after the bolus administration (*p* < 0.05) and, as a result, differed in the parameters calculated during the elimination phase such as the elimination rate constant (K_el_), the half-life of the exogenous glucose load (t_1/2_), and apparent volume of distribution (V_d_; for all, *p* < 0.05). The high dose of Goji supplementation could thus enhance the first-phase glucose-induced insulin secretion. Findings on the offspring were inconsistent and therefore a long-term effect of Goji supplementation during pregnancy could not be demonstrated. Further study on the effect of Goji on the secretory pathway of insulin could clarify its hypoglycaemic action, while different protocols are needed to investigate its potential effects on foetal programming.

## 1. Introduction

Diabetes mellitus is a chronic multifactorial metabolic disorder characterised by an excess of glucose in the blood, known as hyperglycaemia. The incidence of this disease is rapidly and continuously growing worldwide, and it is reported that around 578 million people, corresponding approximately to 10.2% of the world’s population, will be affected by diabetes in 2030 [1]. Hyperglycaemia can be caused by insufficient insulin production due to a loss of the physical or functional β-cell mass (diabetes of type 1, T1D) or insulin resistance, i.e., reduced response to the insulin in the peripheral target tissues (liver, muscles, and adipose tissue; diabetes of type 2, T2D) [2]. Ethnicity, age, overweight, and history of gestational diabetes (GDM) are some proven risk factors for T2D [1,2]. Importantly, offspring born to mothers with diabetes also have an elevated risk to develop T2D, and thus maternal nutrition could play a key role in the development of diabetes [3,4]. This theory, referred to as foetal metabolic programming and Developmental Origins of Health and Disease (DOHaD) [4,5,6], came from the epidemiological studies of Barker et al. which first linked metabolic syndromes to low birth weight [7]. The theory proposed by these authors was then expanded, and studies on DOHaD today are not only about under- or overnourishment but also focus on the presence or deficiency of specific nutritional components during pregnancy and lactation. Patients with diabetes present hyperglycaemia, impaired glucose tolerance, dyslipidaemia, hyperinsulinemia, and/or persistent insulin deficiency [2]. There are many oral drugs available for T2D, although they show several limitations and drawbacks, such as deficiency in multiple dosage regimen, high-cost, adverse side effects, and toxicity [8], while it is now commonly accepted that lifestyle changes in diet and exercise are important strategies for its prevention and control [2,8]. In this context, it is an urgent requirement to explore new strategies to find natural, safer, and more effective hypoglycaemic compounds for the treatment of T2D.

Recently, isolating and researching new ingredients from natural resources with biological activity and beneficial effects on health have attracted a lot of attention. Goji berry, or wolfberry, is the fruit of *Lycium barbarum*, consumed by Tibetans and Chinese over the centuries as a traditional medicinal herb and food supplement [9,10]. Over the last decades, Goji berry has also captured the interest of the Western countries for its numerous potential beneficial effects both on the general well-being of the individual and on the prevention and treatment of several pathologies, including diabetes [9,10]. The berries contain many substances with biological activity in large concentrations such as carotenoids, vitamins (riboflavin, thiamin, and ascorbic acid), flavonoids, and amino acids, with proline as the major constituent [10,11]. However, it seems that polysaccharides (*Lycium barbarum* polysaccharides, LBPs), of which the fruit is particularly rich, are the primary bioactive components to determine the beneficial pharmacological effects of the berry [11,12,13,14,15]. Previous studies have shown that Goji berries have antioxidant, anti-aging [12], antitumoral [16], anti-inflammatory [17,18], immunomodulatory [19], gastrointestinal-protective [20], cardioprotective [13], retinoprotective [14], and neuroprotective properties [21] as well as beneficial effects on several aspects of the reproductive sphere [22,23]. Last but not least, an increasing number of studies are showing the influence of Goji berries on blood glucose level homeostasis, suggesting antidiabetic [15,24,25,26,27] and hypolipidemic effects [26,28,29]. For a review, see Kwok et al. [10] and Masci et al. [11]. These studies were performed both in vivo and in vitro, although the exact mechanisms by which Goji berries induce hypoglycemic effects still need to be elucidated.

Moreover, in these studies, the most commonly used animal models are rodents, while the effects on the rabbit are still little explored [22,30,31]. The rabbit, however, is mainly a livestock species, and it is also a pet as well as an animal model used in experiments and studies regarding insulin resistance [32,33,34], effects of nutraceutical products on glucose homeostasis [35,36,37], pregnancy physiology [38,39,40,41], and foetal metabolic programming [42]. Most importantly, the hormonal profile of the pregnant doe is comparable to that of women, including the condition of insulin resistance [43,44,45]. Menchetti et al. [46] have recently evaluated the effect of Goji berries on the concentration of several hormones of rabbits but samples through pregnancy were not collected, and only simple indices of insulin resistance were used (i.e., fasting plasma glucose and insulin concentrations; Homeostatic Model Assessment for insulin resistance, HOMA). Furthermore, according to the theory of DOHaD, the mother’s nutrition could have long-term effects on the offspring metabolism [3,5]; however, only the short-term effects on rabbit does were investigated by these authors. The present study, on the other hand, hypothesises that supplementation with Goji berries could influence insulin sensitivity of rabbit does not only during pregnancy but also in the adult life of their offspring. Moreover, for the first time, both simple and glucose tolerance test-derived indices were measured in pregnant rabbits and their litters.

Therefore, the aim of this study was to evaluate the effects of a dietary supplementation with two different concentrations of Goji berries, 1% and 3%, during pregnancy on the insulin sensitivity of pregnant rabbit does and their offspring by using simple and intravenous glucose tolerance test-derived indices.

## 2. Materials and Methods 

The experiment was conducted at the farm of the Agricultural University of Tirana, Faculty of Veterinary Medicine, Albania. The animals were maintained in accordance with Legislative Decree No. 146, implementing Directive 98/58/EC regarding the protection of animals that were kept for farming purposes, and were daily checked by the responsible veterinarian of the farm. All efforts were made to minimise animal distress and to use only the number of animals necessary to produce reliable results.

### 2.1. Animals and Experimental Design

Nulliparous New Zealand White rabbits (n = 75) were individually housed in controlled environmental conditions: temperature ranged from +18 to +23 °C, relative humidity ranged from 60% to 75%, and the lighting schedule was 16 L/8 D. Two months before the artificial insemination (AI), the rabbit does were randomly assigned to three dietary groups (n = 25 rabbits/group): control group (C) was fed with commercial standard diet, while G1 and G3 groups with the same feed supplemented with 1% and 3% of Goji berries, respectively. The diets supplied to the does (Table 1) were isoenergetic, iso-nitrogenous, formulated according to current dietary recommendations for the rabbit [47]. These diets have been already used in previous studies [30,46,48,49]. During the adaptation period, the does were provided 150 g/d of the different feed, while the pregnant does were fed ad libitum. Water was always provided ad libitum.

**Figure 1:**
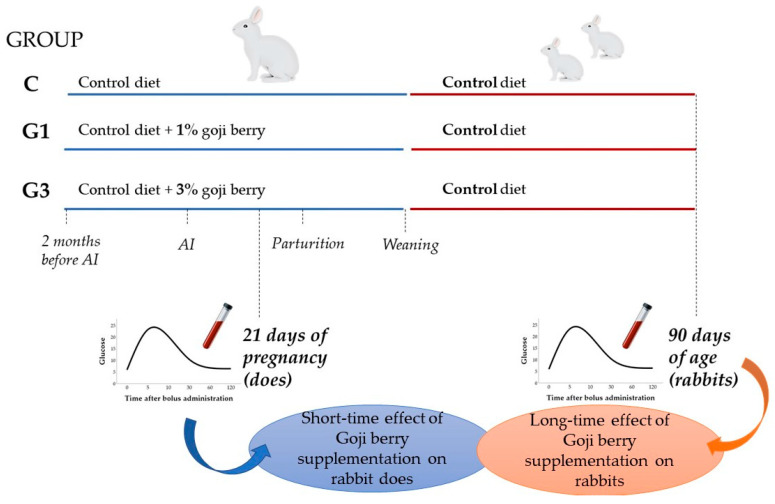
Graphic representation of the experimental protocol. From two months before artificial insemination (AI) until weaning of the litters, rabbit does of groups C, G1, and G3 were fed a standard diet (C group), standard diet supplemented with 1% Goji berry (G1 group), and 3% Goji berry (G3 group), respectively (n = 75). After weaning, rabbits in all groups received the standard diet. Simple and intravenous glucose tolerance test-derived indices were estimated at 21 days of pregnancy on rabbit does to evaluate the short-time effect during pregnancy, and at 90 days of age on rabbits to evaluate the long-term effects on the offspring. The blue and red lines indicate the maternal and offspring feeding, respectively.

Ovulation was induced by injecting 0.8 μg of synthetic GnRH (Receptal, Hoechst-Roussel Vet, Milan, Italy) just before AI [51]. Artificial insemination was performed with 0.5 mL of diluted fresh semen. Pregnancy was diagnosed by manual palpation 12 days after AI, and non-pregnant does were excluded from the trial (number of rabbits excluded as non-pregnant = 7, 5, and 8 rabbits in C, G1, and G3 groups, respectively). Fertility rates were 0.72, 0.80, and 0.68, whereas litter sizes at birth were 8, 7, and 7 rabbits (standard error = 1 for all) for C, G1, and G3 groups, respectively. Lactation was controlled until day 20 post-partum by opening the nest once a day for 5–10 min. After the 20th day of lactation, the nest was opened, and the young rabbits had free access to the mother’s diets. At weaning (day 35 post-partum), they were moved to individual cages and fed a control diet. The nomenclature of the offspring groups was maintained in accordance with the nutritional treatment of the mothers (C, G1, and G3 groups). Indices of insulin resistance were estimated at 21 days of pregnancy on rabbit does to evaluate the effect of Goji supplementation during pregnancy, as well as at 90 days of age on rabbits to assess whether the mother’s nutrition could have long-term effects on the offspring (Figure 1).

During the trial, feed intake of does and their offspring was recorded daily, while body weight (BW) was measured weekly, between 8:00 and 10:00 a.m. 

### 2.2. Indices of Insulin Resistance

The insulin resistance assessment methods adopted in this study were classified into simple indices that require a single blood sampling performed with the fasting animal and does not require administration of exogenous glucose, as well as dynamic tests where samples are collected in series after glucose administration [52].

#### 2.2.1. Simple Insulin Resistance Indices with Fasting Sampling

Blood samples (about 1 mL) were collected from the marginal ear vein into tubes containing EDTA at day 21 of pregnancy in rabbit does (n = 13/group randomly selected) and at day 90 of age in the offspring (n = 13/group randomly selected female rabbits born to the does evaluated during pregnancy) between 8:00 and 10:00 a.m, after fasting for at least 16 h [45]. Animals were selected using the RANDBETWEEN function of Excel. Samples were immediately centrifuged at 3000 × *g* for 15 min, and plasma was stored frozen until assayed for glucose and insulin. Plasma insulin concentrations were determined using RIA procedures as previously described [43,53]. Briefly, plasma insulin was determined by the double antibody/PEG technique using a porcine insulin RIA kit (Linco Research Inc. St Charles, MO, USA). The antiserum was guinea pig anti-porcine insulin, while both labelled and standard antigen used purified recombinant human insulin. The limit of sensitivity was 2 μU/mL, and intra- and inter-assay coefficients of variations were 6.8% and 9.2%, respectively. The glucose concentrations were analysed according to García-García et al. [54] using the glucose oxidase method by the Glucose Infinity kit from Sigma (Sigma Diagnostic Inc., St. Louis, MO, USA). 

Glucose and insulin concentration were used to calculate the following indices: fasting plasma glucose (in mmol/L) and insulin (in µU/mL) concentrations; glucose/insulin ratio (G/I); Homeostatic Model Assessment (HOMA) for insulin resistance index was calculated as [insulin concentration × (glucose concentration ⁄18)] ⁄22.5 [43,45]. Lower HOMA-IR values indicate a relatively greater insulin sensitivity.

#### 2.2.2. Intravenous Glucose Tolerance Test and Determination of Kinetic Parameters of Glucose

Intravenous glucose tolerance tests (IVGTT) were performed on 7 randomly selected animals/group of each category (pregnant does 21 days after AI and 90-day-old rabbits) between 8:00 and 10:00 a.m, after fasting for at least 16 h, as previously reported [45]. Animals were selected using the RANDBETWEEN function of Excel among those already chosen for the fasting test. Moreover, the 90 day selection, was restricted to females born to the rabbit does evaluated during pregnancy. A single bolus of glucose (0.6 g/kg of body weight) was rapidly infused into the ear marginal vein through an 18G catheter. A small drop of capillary blood was collected by puncturing the ear just before glucose administration and subsequently after 5, 10, 30, 60, and 120 min. Blood glucose (mmol/L) was measured with a calibrated glucometer (OneTouch^®^UltraEasy, LifeScan Europe, Johnson and Johnson, Zug, Switzerland), using test strips from the same supplier. The inter-assay coefficient of variation for glucose was <5%. The glucose concentrations at these time points were used to calculate kinetic parameters using an open mono-compartmental model as previously reported [45]. The elimination rate constant (K_el_) was calculated as the slope of the semi-logarithmic curve of the glucose concentration with respect to time during the elimination phase. The half-life (t_½_) of the exogenous glucose load was obtained as (ln 2/elimination rate constant), the apparent volume of distribution (V_d_) was obtained as a ratio between the dose and the glucose concentrations after administration of the bolus, while clearance (CL) was obtained as the volume of distribution X the rate of elimination constant. The area under the concentration-time curve (AUC) was calculated using the linear trapezoidal method using GraphPad Prism software version 5.01 (GraphPad Software, San Diego, CA, USA).

### 2.3. Statistical Analysis

Assumptions and outliers were checked using diagnostic graphs and Levene’s test. Glucose-to-Insulin Ratio was log-transformed to improve data distribution. Differences between groups (3 levels: C, G1, and G3) in mean BW and indices of insulin sensitivity were assessed using the one-way analysis of the variance (ANOVA). Sidak correction was used for multiple comparisons. ANOVA with the Welch statistic and Games-Howell for multiple comparisons were used to analyse G/I and Vd as Levene’s tests indicated unequal variances among groups. Dynamic changes in glucose levels after bolus administration included repeated measurements and were thus investigated using linear mixed models. These models evaluated the effect of the animal’s category (2 levels: pregnant does and 90-day-old rabbits), time (6 levels: from 0 to 120 min after bolus administration), group (3 levels: C, G1, and G3), and their interaction. In these models, the animals were treated as a subjects variable while the time was treated as a repeated factor. Sidak adjustment was used for multiple comparisons. Finally, Pearson correlation coefficient was used to assess whether BW was associated with insulin resistance indices in rabbit does and 90-day-old rabbits, regardless of nutritional treatment. The correlation was considered poor if r < │0.3│, medium if │0.3│ ≤ r < │0.5│, and large if r ≥ │0.5│ [55]. Statistical analyses were performed with SPSS Statistics version 25 (IBM, SPSS Inc., Chicago, IL, USA). A result with *p* ≤ 0.05 was defined as significant, with *p* between 0.1 and 0.5 it was defined as a trend.

## 3. Results

### 3.1. Body Weight

Nutritional treatment did not affect the BW of pregnant rabbits (*p* = 0.201), while differences were found in 90-day-old rabbits (*p* < 0.001). In particular, rabbits of the G1 group had the highest BW (*p* < 0.01).

### 3.2. Fasting Blood Sample-Based Indices

Table 2 shows simple indices of insulin resistance in pregnant does and 90-day-old rabbits according to the nutritional group. Fasting plasma glucose concentration ranged from 5.11 to 27.78 mmol/L in pregnant does, and from 5.44 to 23.06 mmol/L in 90-day-old rabbits, while insulin concentrations ranged from 2.08 to 14.24 μU/mL in rabbit does, and from 4.6 to 12.10 μU/mL in rabbits. Greater variability was also found in pregnant animals for the G/I values, ranging from 0.41 to 3.21 in does, and from 0.61 to 1.33 in 90-day-old rabbits. Differences between groups in mean values of these parameters could not be found in any category of animal; however, the HOMA index of 90-day-old rabbits was higher in G3 than C group (*p* < 0.05).

### 3.3. Changes after Bolus Administration and IVGTT Derived Indices

**Figure 2:**
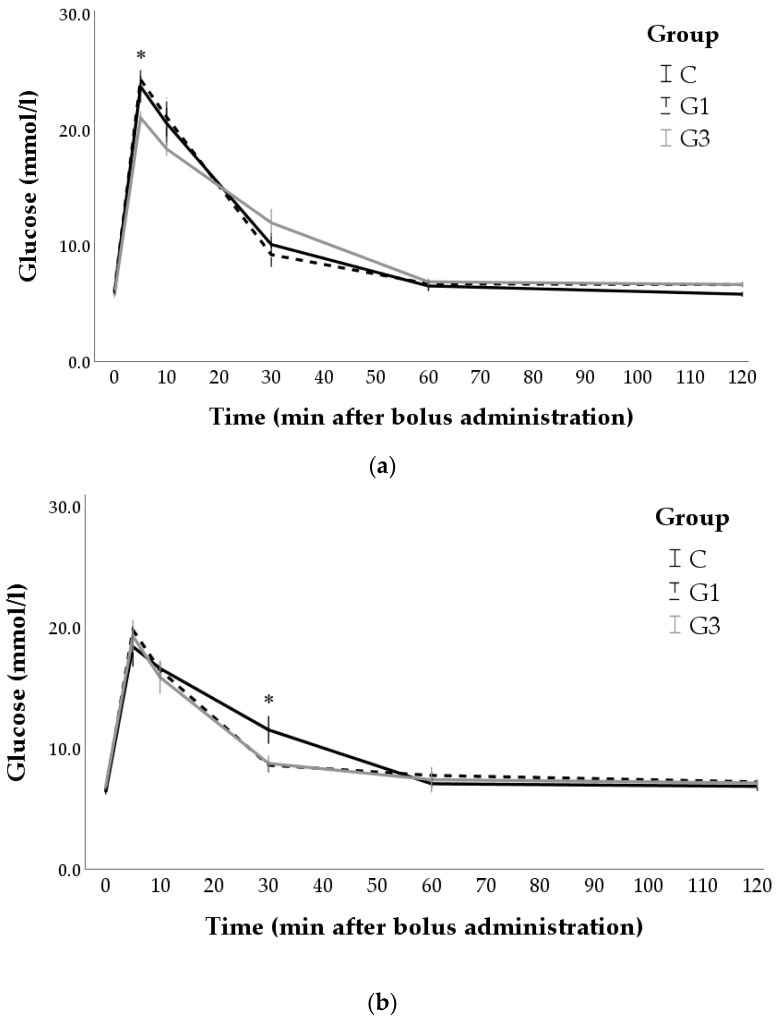
Changes in blood glucose levels after bolus administration in pregnant does (**a**) and 90-day-old rabbits (**b**) of control group (C, n = 7/category) and groups with 1% (G1, n = 7/category) or 3% (G3, n = 7/category) of Goji berry. * *p* < 0.05: G3 vs. C group.

Figure 2 shows the profiles for glucose disappearance following glucose administration in pregnant does and 90-day-old rabbits. Glucose levels were influenced by the category of animals as the marginal means were higher in pregnant does than in 90-day-old rabbits (11.95 ± 0.20 and 11.13 ± 0.19 mmol/L in does and 90d rabbits, respectively; *p* = 0.003). Regardless of nutritional treatment, glucose levels peaked as early as 5 min after bolus administration and returned to baseline after 60 min in both pregnant females and 90-day-old rabbits (*p* < 0.01). Multiple comparisons showed that pregnant does belonging to G3 group had the lowest values in early sampling (i.e., 5 min after injection; *p* < 0.05). When compared with G1 group, G3 group also had lower concentrations at 10 min (*p* < 0.05) and higher at 30 min (*p* < 0.05) after bolus administration. Regarding 90-day-old rabbits, differences were found at 30 min, when C group showed higher values compared to the two groups receiving Goji supplementation (*p* < 0.05).

Regarding glucose tolerance test-derived indices (Table 3), several differences between groups were found in pregnant does, which mainly involved the G3 group. Indeed, it showed lower mean values of C_max_ (*p* < 0.05) and K_el_ (*p* < 0.05) but higher V_d_ (*p* < 0.05) than G1 group. The t_1/2_ tended to be higher in G3 than C and G1 groups (*p* < 0.1). Conversely, no significant differences were found in the glucose tolerance test-derived indices of 90-day-old rabbits.

### 3.4. Correlations between Body Weight and Indices of Insulin Resistance 

The Pearson coefficient showed a significant correlation only between BW and AUC in pregnant rabbits (*p* < 0.05; Table 4).

## 4. Discussion

To the best of our knowledge, this is the first study to estimate the effects of Goji berries on insulin resistance during pregnancy as well as to investigate the possible effects of maternal Goji berry intake on the adult offspring. The rabbit was used as an experimental model, and both simple (i.e., fasting glycemia, insulinemia, and HOMA-IR levels) and glucose tolerance tests indices were determined. In the present study, Goji berries modulated the response to glucose load of pregnant females, while long-term effects on the offspring were not found.

*Lycium barbarum* is also well known in traditional Chinese herbal medicine for its beneficial effects on glucose metabolism. Few scientific studies, however, have investigated this, and most of them used rodents with induced diabetes as an experimental model [25,27,56,57]. To our knowledge, only one study has used the rabbit [26], while the effects of Goji berries during pregnancy and on the adult offspring have never been explored. Thus, although our hypotheses were only partially confirmed, the present study maintains the innovative aspects due to the experimental model. Compared to rodents, the rabbit was suitable to verify our hypotheses because its dimensions facilitated the execution of the IVGTT. The IVGTT is a very accurate test which is able to detect abnormalities prior to diabetes onset [58] and is considered a reference technique for epidemiological and physiological studies [52], although it is more time and resource-consuming than fasting sampling. On the other hand, fasting-derived indices were simple and practical. They are the most commonly used indices of insulin resistance although they can mainly indicate hepatic insulin resistance [52], i.e., impaired suppression of glucose production by insulin in hepatocytes [59]. Our results did not show alterations or differences between groups in the values of the fasting indices, suggesting that no nutritional treatment induced pathological conditions of hepatic insulin resistance. Conversely, several differences were found in response to IVGTT. This finding could indicate that a single fasting sample may not be sensitive enough to capture differences in healthy rabbits, while IVGTT had a higher sensitivity to changes in the diet. In agreement with our findings, it has been shown in humans that abnormalities of the IVGTT are more sensitive risk factors for T1D than HOMA [58]. In particular, the first-phase glucose-induced insulin secretion appears to be an important determinant of glucose tolerance [58,60]. The response to an IVGTT is, in fact, biphasic: an early peak of insulin, detectable after 5 min, derives from the reserves already stored in the β-cells, while the second peak, after 20–30 min, is due to the de novo synthesis of insulin by the β-cells [61]. This bimodal curve has not been clearly demonstrated in the rabbit, although it is likely that in healthy animals, the sequence is similar as the insulin concentration peaks after 5 min and appears also high 30 min after the glucose injection [33,34].

The main results of this study seem indeed to be related to the first-phase glucose-induced insulin secretion. An effect of Goji berry supplementation was found on pregnant rabbits, and it was mainly linked to the glucose dynamics of the first minutes after the administration of the bolus. Does supplemented with the highest concentration of Goji berries (G3 group) had the lowest glucose concentrations 5 min after the injection. In particular, maximum glucose concentration of G3 was reduced by 12.3% compared to the control group, and by 17.1% compared to G1 group. Therefore, a high dose of Goji supplementation seemed to enhance the early insulin release from the β-cells. This finding suggested that Goji berry could act on mechanisms that regulate the secretory pathways of the insulin stored in the β-granules already docked at the plasma membrane of the β-cell or in the number of mature insulin granules. Thus, further studies which also determine the dynamic changes in insulin or focus on β-cell insulin stores could clarify the mechanisms involved in the hypoglycaemic action of Goji berry. As mentioned previously, this first phase of insulin secretion appears to play an important role in the diagnosis of insulin resistance. In humans, the enhancement of the early phase of insulin secretion improves glucose tolerance [60]. Conversely, the first-phase glucose-induced insulin secretion is reduced in individuals prior to the development of diabetes [58], and it is more or less absent in those with full-blown disease [60]. The profiles for glucose disappearance following the first peak seems to suggest that the supplementation did not influence the second phase of insulin secretion in pregnant does. After 60 min from the administration of the bolus, in fact, the values returned to baseline in all rabbits. Moreover, differences in the kinetic parameters such as K_el_ and t_1/2_ could only be a consequence of the maximum concentrations reached by the three groups. These parameters were obtained from regression analyses during the glucose elimination phase. Thus, it was logical to expect a lower elimination rate and a longer half-life in the G3 group because its glucose concentrations started from a lower peak and arrived at the same final value as the other rabbits. However, it is interesting to note that the maximum glucose concentrations as well as kinetic parameters of low-dose group (G1 group) did not differ from the control group, indicating a dose-dependent effect of Goji berry supplementation.

To date, only a clinical study would prove the efficacy of Goji berry supplementation in T2D patients [24], although several experimental studies have yielded encouraging results. The hypoglycaemic effect of Goji berries was indeed found in rodents in the following studies: fed with a high-fat diet [29], streptozotocin-induced diabetes [25], alloxan [27], and genetic mutations [15]. The rabbit was adopted as an experimental model only by Luo et al. [26] using alloxan-induced diabetic and hyperlipidaemic animals. To our knowledge, Goji berry supplementation during pregnancy has never been investigated in any animal model. Therefore, our idea was to use not only a poorly investigated animal model but also a condition of insulin resistance not yet explored. Over the course of normal pregnancy in women, insulin sensitivity physiologically decreases to limit maternal glucose utilisation and divert glucose toward the growing foetus [4,46]. However, when β-cells of the pregnant woman are unable to produce an adequate amount of insulin to face this increased demand, the physiological reduction in insulin sensitivity becomes a pathological condition, i.e., the gestational diabetes mellitus (GDM). The GDM prevalence has increased worldwide and its associations with subsequent T2D and other diseases have been demonstrated [3,62]. In addition to the Goji berries as a nutraceutical to improve post-prandial glucose, the present study proposed the rabbit doe as a model to study physiological and pathological insulin resistance changes during pregnancy. Two results support this choice. First, pregnant rabbits had mean values of glucose during IVGTT higher than those of the 90-day-old rabbits. This result may indicate that insulin sensitivity decreases during pregnancy in rabbits (as in women). It confirms previous studies [43,44,45], although the different ages of the two animals’ categories could represent a bias. Second, the body weight of the does was positively correlated with the glucose AUC, indicating that insulin resistance enhances as the weight of pregnant rabbits increases. This finding confirms that being overweight worsens insulin sensitivity during pregnancy, as widely demonstrated in women [4,62]. Thus, further studies could confirm the hypoglycaemic effect of Goji berries in rabbit does with the prospect of their use during human pregnancy, especially for women predisposed to GDM.

Another main hypothesis of this study was that maternal supplementation with Goji berries could influence insulin sensitivity later in life. To verify this, we fed all the litters a control diet after weaning, and no intervention was applied. They only kept the nomenclature in accordance with the nutritional treatment of their mothers. Thus, any differences between groups in insulin resistance indices of the offspring could be attributed to early life experiences, which only differed in Goji supplementation. Goji berries are rich in carbohydrates, amino acids, vitamins, and polyphenols, and it has recently been shown that these components could play a key role on foetal metabolic programming [6]. The maternal diet seems to program the predisposition to metabolic syndrome, including insulin resistance and glucose intolerance, mainly through epigenetic alterations as they are sensitive to environmental factors such as nutrition, particularly during early life [3,5,6]. In the present study, however, the differences in insulin resistance indices found in the offspring were too weak to support our hypothesis. Further studies could increase the sample size and evaluate the effects of a higher dose of Goji berries in the maternal diet, the inclusion of the berries in the diet also after weaning, or the longer-term consequences.

## 5. Conclusions

Our study confirmed the validity of the rabbit as an animal model for insulin resistance studies because the intravenous glucose tolerance test resulted feasible and sensitive to nutritional treatment. Moreover, for the first time, this test was performed during rabbit pregnancy, a condition of low insulin sensitivity that is physiological and not drug-induced. Goji berry supplementation modulated the response to glucose load in rabbit does, mainly influencing the early phase of insulin secretion. Investigations focused on the secretion of the insulin already docked at the plasma membrane of the β-cell could therefore clarify the mechanisms of the hypoglycaemic action of Goji berry. This effect was dose-dependent, as only the 3% of Goji inclusion could reduce glucose concentration after bolus administration. No effect of maternal diet was instead found in the offspring. Thus, our study did not support a long-term effect of the maternal supplementation in the offspring, although foetal programming deserves further study by using different diets and experimental protocols.

## Figures and Tables

**Table 1:** Formulation and chemical composition (as fed) of control (C) and experimental diets supplemented with 1% (G1) and 3% (G3) Goji berries.

	Diet		
	C	G1	G3
**Ingredients** ^1^			
Wheat bran	30.0	29.5	29.0
Dehydrated alfalfa meal	42.0	41.5	41.0
Barley	9.5	9.5	9.0
Sunflower meal	4.5	4.5	4.2
Rice bran	4.0	4.0	3.9
Soybean meal	4.0	4.0	3.9
Calcium carbonate	2.2	2.2	2.2
Cane molasses	2.0	2.0	2.0
Dicalcium phosphate	0.7	0.7	0.7
Vitamin–mineral premix ^2^	0.4	0.4	0.4
Soybean oil	0.4	0.4	0.4
Salt	0.3	0.3	0.3
Goji berries	-	1.0	3.0
**Analytical data** ^1^			
Crude protein	15.74	15.64	15.66
Ether extract	2.25	2.23	2.47
Ash	9.28	9.36	9.25
Starch	16.86	17.07	16.99
NDF	38.05	38.55	37.49
ADF	19.54	19.60	19.01
ADL	4.01	4.31	3.98
**Digestible Energy** ^3^	2464	2463	2459

^1^ as percentage (%). ^2^ Per kg diet: vitamin A 11,000 IU; vitamin D3 2000 IU; vitamin B1 2.5 mg; vitamin B2 4 mg; vitamin B6 1.25 mg; vitamin B12 0.01 mg; alpha-tocopherol acetate 50 mg; biotine 0.06 mg; vitamin K 2.5 mg; niacin 15 mg; folic acid 0.30 mg; D-pantothenic acid 10 mg; choline 600 mg; Mn 60 mg; Fe 50 mg; Zn 15 mg; I 0.5 mg; Co 0.5 mg. ^3^ as Kcal/kg. Estimated by Maertens et al. [50].

**Table 2:** Fasting derived indices of insulin resistance in pregnant does and 90-day-old rabbits of control group (C, n = 13/category) and groups with 1% (G1, n = 13/category) and 3% (G3, n = 13/category) of Goji berry.

Animal Category	Parameter	Group			RMSE	*p*-Value
		C	G1	G3		
Pregnant does	Fasting glucose (mmol/L)	5.88	6.01	5.73	0.48	0.457
	Fasting insulin (μU/mL)	7.32	6.50	5.86	3.30	0.648
	Glucose-to-insulin ratio *	0.96	1.25	1.30	0.67	0.632
	HOMA-IR *	0.11	0.09	0.09	0.01	0.567
90-day-old rabbits	Fasting glucose (mmol/L)	6.36	6.50	6.80	0.61	0.271
	Fasting insulin (μU/mL)	7.47	8.03	9.32	1.86	0.094
	Glucose-to-insulin ratio *	0.92	0.86	0.73	0.21	0.594
	HOMA-IR *	0.12 ^b^	0.13 ^a,b^	0.16 ^a^	0.01	**0.037**

* log transformed. HOMA-IR: Homeostatic Model Assessment for insulin resistance. RMSE: root mean square error. Significant *p*-values are in bold (F test, *p* < 0.05). ^a^, ^b^: Means sharing the same superscript are not significantly different from each other for *p* < 0.05 (Sidak correction).

**Table 3:** Intravenous glucose tolerance test-derived indices obtained in pregnant does and 90-day-old rabbits of control group (C, n = 7/category) and groups with 1% (G1, n = 7/category) and 3% (G3, n = 7/category) of Goji berry.

Animal Category	Parameter	Group			RMSE	*p*-Value
		C	G1	G3		
Pregnant does	C_max_ (mmol/L)	23.80 ^a^	24.91 ^a^	20.84 ^b^	2.21	**0.019**
	Glucose 60 min (mmol/L)	6.53	6.72	6.89	0.96	0.862
	AUC ((mmol/L) × min)	1111.93	1133.50	1158.60	115.84	0.802
	K_el_ (%/min)	1.03 ^a^	1.05 ^a^	0.86 ^b^	0.10	**0.018**
	t_1/2_ (min)	68.52 ^B^	66.85 ^B^	79.11 ^A^	7.75	**0.047**
	V_d_ (dl/kg)	1.46 ^a,b^	1.37 ^b^	1.59 ^a^	0.14	**0.023**
	CL (dl/min/kg)	1.45	1.43	1.39	0.18	0.837
90-day-old rabbits	C_max_ (mmol/L)	18.81	18.51	19.26	2.92	0.904
	Glucose 60 min (mmol/L)	7.06	7.76	7.40	1.59	0.794
	AUC ((mmol/L) × min)	1126.28	1101.67	1076.77	110.70	0.745
	K_el_ (%/min)	0.75	0.74	0.76	0.15	0.959
	t_1/2_ (min)	93.73	97.13	85.10	13.00	0.326
	V_d_ (dl/kg)	1.91	1.71	1.71	0.31	0.681
	CL (dl/min/kg)	1.41	1.25	1.31	0.32	0.732

C_max_: maximum concentration of glucose (5 min after bolus administration); AUC: area under the concentration–time curve; K_el_: elimination rate constant; t_1/2_: half-life of the exogenous glucose load; V_d_: apparent volume of distribution; CL: clearance; RMSE: root mean square error. Significant *p*-values are in bold (F test, *p* < 0.05). ^a^, ^b^: Means sharing the same superscript are not significantly different from each other for *p* < 0.05 (Sidak correction, Games-Howell for V_d_). ^A^, ^B^: Means sharing the same superscript are not significantly different from each other for *p* < 0.1 (Sidak correction).

**Table 4:** Pearson coefficient showing correlations between BW and indices of insulin resistance in pregnant rabbit does and 90-day-old rabbits, regardless of group.

Parameter	Animal Category	
	Pregnant Does	90-Day-Old Rabbits
Fasting glucose	0.022	0.281
Fasting insulin	−0.137	−0.054
Glucose–insulin ratio ^#^	0.109	0.166
HOMA	−0.105	0.024
C_max_	0.153	0.060
Glucose 60 min	0.158	0.165
AUC	0.539 *	0.248
K_el_	−0.116	0.071
t_1/2_	−0.033	0.254
V_d_^#^	−0.266	−0.290
CL	−0.202	−0.119

^#^ after log-transformation. * Correlation is significant at the 0.05 level (two-tailed), in bold. C_max_: maximum concentration of glucose (5 min after bolus administration); AUC: area under the concentration-time curve; K_el_: t_1/2_: V_d_: apparent volume of distribution; CL: clearance

## Data Availability

The data presented in this study are available in the article. Further information is available upon request from the corresponding author.
